# A Backscatter Suppressed Electron Detector for the Measurement of “a”

**DOI:** 10.6028/jres.110.066

**Published:** 2005-08-01

**Authors:** A. Komives, F. E. Wietfeldt, C. Trull, F. B. Bateman, M. S. Dewey, A. K. Thompson, R. Anderman, S. Balashov, Yu. Mostovoy

**Affiliations:** DePauw University, Greencastle, IN 46135; Tulane University, New Orleans, LA 70118; National Institute of Standards and Technology, Gaithersburg, MD 20899; Hamilton College, Clinton, NY 13323; Kurchatov Institute, Moscow, Russia

**Keywords:** beta decay, electron spectroscopy, neutron decay, scintillation detectors

## Abstract

A new method of measuring the electron-antineutrino angular correlation coefficient, little “*a*”, from neutron decay—to be performed at the National Institute of Standards and Technology—will require an electron spectrometer that strongly suppresses backscattered electrons. A prototype consisting of six trapezoidal veto detectors arranged around a plastic scintillator has been tested with an electron beam produced by a Van de Graaff accelerator. The results of this test and its implications for the little “*a*” measurement are discussed.

## 1. Introduction

**Fig. 1:**
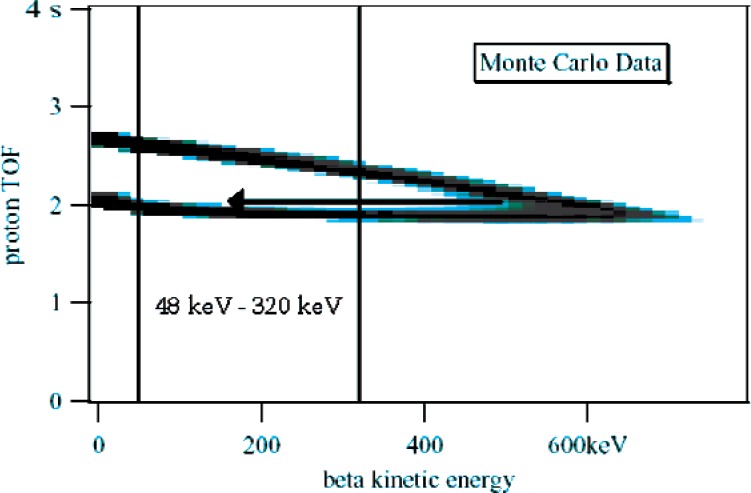
For decays with electron energies in the range 300 keV to 782 keV, backscattered electrons from the upper arm will be measured with a lower energy, as suggested by the arrow, and thus can be identified with the lower arm resulting in a systematic error.

Little “*a*” is the momentum correlation coefficient of the electron and antineutrino produced in neutron beta decay. One among several correlation coefficients describing this decay, little “*a*” has the largest uncertainty, 4 %. Increasing the precision of correlation coefficient measurements provides ever more stringent tests of the electroweak Standard Model. A new technique for measuring little “*a*” involves measuring the time-of-flight (TOF) of the proton using the corresponding decay from the electron as a start signal [1] [2] [3] [4]. The resulting proton TOF vs. electron energy plot for numerous decays, produced by a Monte Carlo calculation, is shown in Fig. 1. As can be seen by the arrow, electrons belonging in the upper TOF arm could appear in the lower arm by backscattering off the electron detector before the total energy is deposited. Because little “*a*” is related to the difference in the number of events between the two TOF arms, this mislabeling will result in a systematic uncertainty. Further Monte Carlo calculations using ETRAN [5] show a 0.5 % measurement of little “*a*” will require rejection efficiency greater than 80 % for the backscattered electrons.

## 2. Prototype Detector Design

**Fig. 2:**
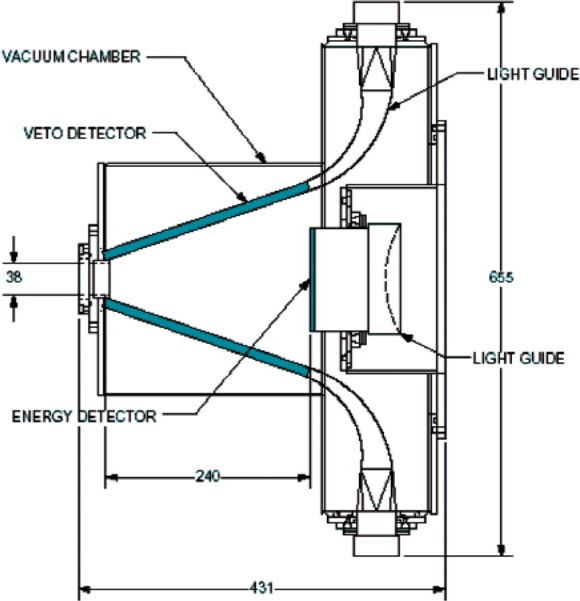
The energy and veto detectors, with light guides, arranged inside the vacuum chamber. The photomultiplier tubes are not shown. All dimensions are in millimeters.

**Fig. 3:**
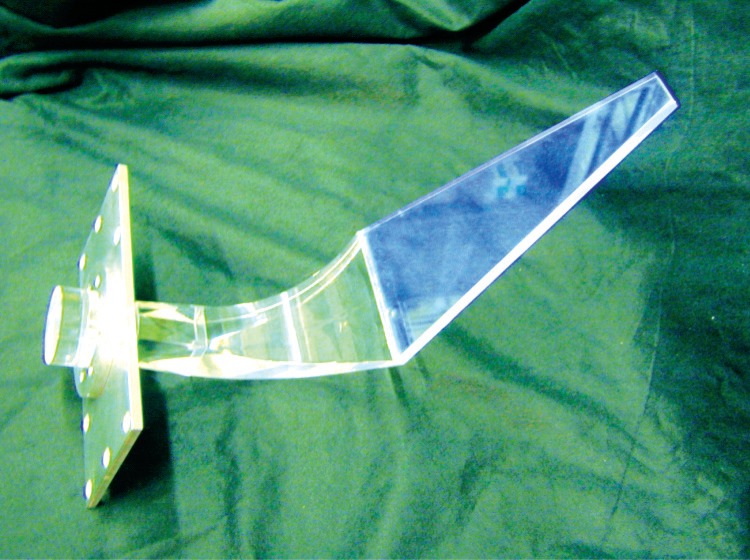
One of the veto paddles attached to its light guide. Six of these paddles were assembled together into a close-fit hexagonal cone.

The design of choice was a 5.0 mm thick 120.7 mm diameter cylindrical Bicron^1^ BC-408 plastic scintillator energy detector surrounded by six trapezoidal veto scintillator paddles, made from the same plastic, in a conical arrangement as shown in Fig. 2. An event from a veto paddle in coincidence with the energy detector tags the backscattered electron. Figure 3 shows one of the six veto paddles from the prototype attached to a Polycast UVT acrylic light guide. Likewise, the energy detector was also attached to a cylindrical light guide of the same material. For five of the veto detector light guides a Burle 8575 2 inch photomultiplier tube (PMT) was attached. For comparison purposes, the sixth light guide was affixed to a Burle 8850 2 inch PMT which has a higher gain first dynode. The energy detector’s light guide was attached to a Burle 8854 5 inch PMT. The veto and energy detector PMT’s ran at −2900 V and −1770 V, respectively.

The energy detector produced an energy signal that was analyzed by a charge-integrating analog-to-digital converter (QDC) and a timing signal used to produce an event trigger. The event trigger, in turn, was used to gate the QDC, produce a start for the time-to-digital converter (TDC), and trigger a 1 ms gate that inhibited other incoming events until the acquisition system had time to convert and readout the signals. Each veto detector also produced an energy signal for the QDC and a timing signal that stopped the TDC. Examining the start and stop TDC signals from the energy detector/veto paddle combination allowed the identification of backscattered events. The data acquisition system was controlled by a desktop PC via a CAMAC interface.

## 3. Electron Beam Tests

A 1 MeV beam of electrons, produced by the NIST Van de Graaff accelerator facility, was used to test the prototype which was placed at the end of the electron beam pipe. The 25 micro meter Kapton window on the exit aperture of the beam pipe, an identical Kapton window on the entrance aperture of the detector, and a 155 mm air gap between these two windows degraded the electron energy to 976 keV. A knife edge Pb collimator was placed in front of the entrance window to reduce the likelihood of electron scattering upon entering the detector assembly which was pumped out to 10 Pa. The beam current was less than 100 nA during the tests as higher currents tended to overwhelm the data acquisition system.

**Fig. 4:**
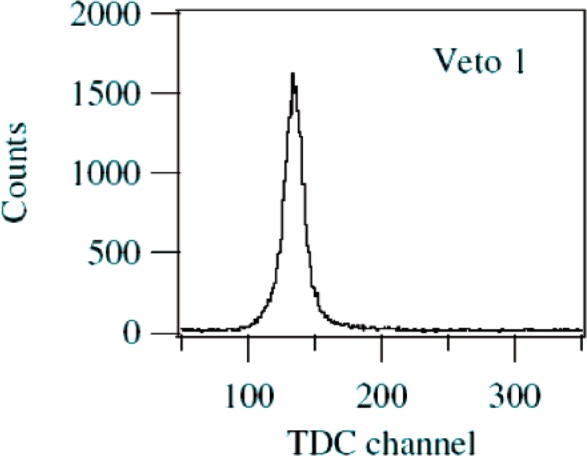
The TDC spectrum for veto detector 1.

**Fig. 5:**
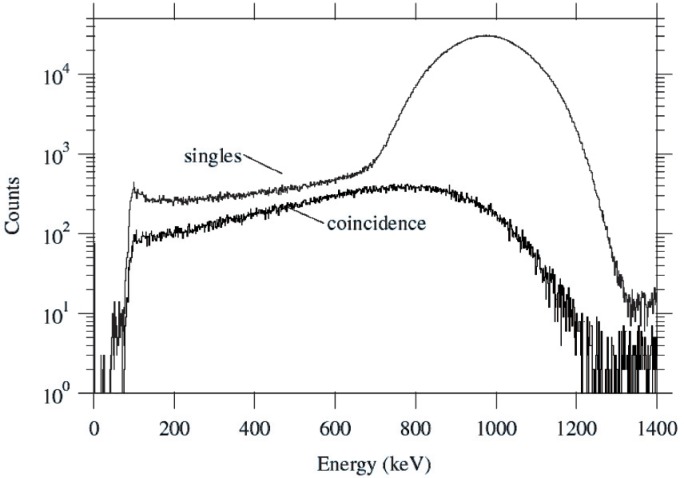
Energy detector singles events, and coincidence events with one or more veto detectors. The low energy tail in the singles data amounts to 4 % of the total events seen, whereas 2.8 % of the total are contained in the coincident data. The missing 1.2 % are thought to be due to electrons losing energy via scattering on beamline components and bremsstrahlung emission before encountering the energy detector.

The analyzed data represents 7200 s (real time) of beam at an average electron singles count rate of 830 s^−1^. Figure 4 illustrates a TDC timing spectrum from veto detector number 1. The other detectors produced similar looking TDC spectra. The events in the energy detector that were coincident with events in the peak of the TDC spectra from one or more veto detectors are shown in Fig. 5. Also displayed are all events recorded by the energy detector. These single events indicate an energy resolution of 26 % full width at half maximum (FWHM) with a non-Gaussian low-energy tail amounting to about 4 % of the total. The coincidence spectrum accounts for 2.8 % of the total number of single events. Monte Carlo calculations indicate this amount should be 1.8 %, with the difference primarily thought to be due to events that involve electron scattering from inside the knife edge collimator to a veto detector, from which it is scattered into the energy detector. Such events were not included in the Monte Carlo simulation and, furthermore, cannot be distinguished with the data collected from events in which an electron scattered from the energy detector into the veto detector. Another possible effect, which was also not a part of the calculation, involves bremsstrahlung photons detected by a veto detector produced by electrons hitting the energy detector. However, calculations determine less than 0.3 % of veto coincidences would be caused by this effect making it a minor consideration. The difference between the 4 % tail seen in the singles data and the 2.8 % in the coincidence data must come from events that involve energy loss before reaching the energy detector. In setting up the detector on the beamline, it was observed the shape of the singles tail was highly dependent on beam conditions and the position of shielding around the detector. The data in Fig. 5 show the smallest tail achieved during our run. Accordingly, it is believed the singles tail contains electrons which have backscattered on the beamline elements and/or x rays produced by such events.

**Fig. 6:**
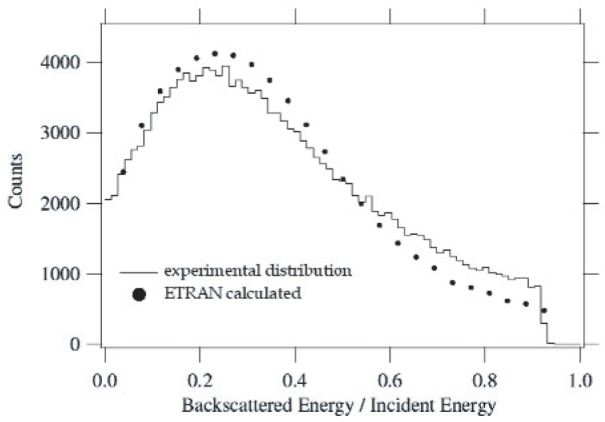
The distribution of ratio of backscattered energy to incident energy measured by the missing energy in the energy detector (solid) and the calculated distribution using the ETRAN Monte Carlo on plastic scintillator (dots). The calculated distribution has been convoluted with a Gaussian to simulate the energy resolution of the detector.

Figure 6 shows the energy of the backscattered electrons determined by subtracting the energy deposited in the energy detector from the beam energy and compares it to an ETRAN Monte Carlo calculation convo- luted with a Gaussian to account for the energy resolution of the detector. The discrepancy for electrons that backscatter with more than 50 % of their incident energy may be caused by these higher energy electrons having a greater probability of producing at least one photoelectron in a veto detector. Otherwise the agreement between data and calculation is quite good.

**Fig. 7:**
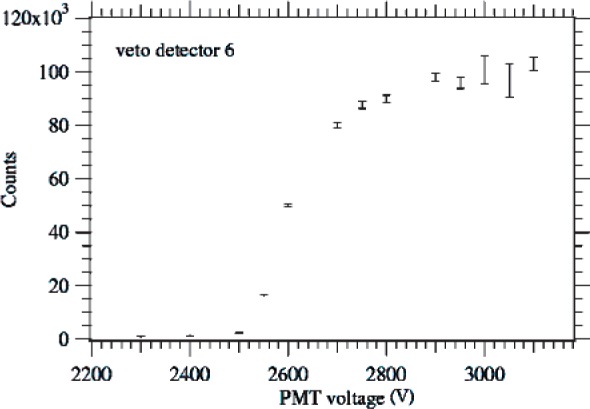
The number of single photoelectron events detected using a visible light source and a small light leak in the detector for various PMT base voltages taken without an electron beam.

An important characteristic of the detector system is the efficiency of the veto paddles for detecting a backscattered electron. The first step in determining this was to use a tiny light leak in the entrance aperture of the detector to measure the efficiency for detecting single photoelectron events. These measurements were done with the electron beam off and involved counting pulses from the veto paddle PMTs for a specified length of time. The entrance aperture was then covered with a light-tight shield and the pulses counted for the same interval to subtract the ever-present dark current. This sequence was then repeated for a series of PMT base voltages. Figure 7 shows the resulting data for veto detector 6. This curve is effectively the integral of the single photoelectron pulse height distribution (approximately a Gaussian). At a PMT voltage of 2500 V the discriminator threshold is above the entire single photoelectron distribution. At about 2600 V the threshold is close to the peak of the distribution. At 2900 V the threshold is below the distribution and the single photoelectron counting efficiency remains constant as the PMT voltage is increased further. When the electron beam data were taken, the veto detector PMT voltages were 2900 V, so the single photoelectron counting efficiency was effectively 100 %.

**Fig. 8:**
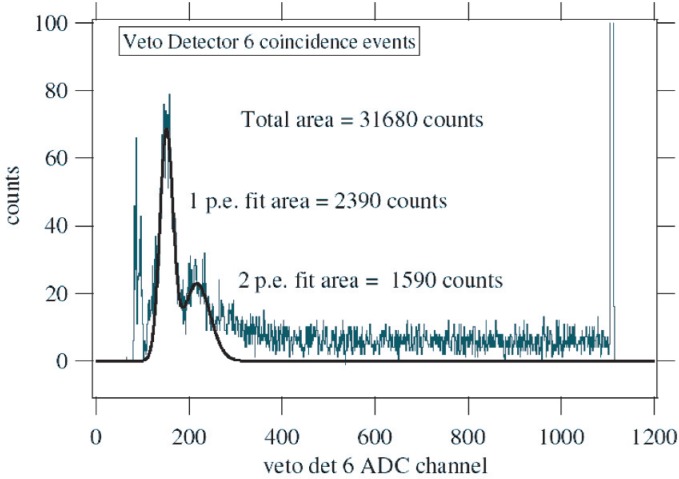
The pulse height (ADC) spectrum of veto detector 6 for events in coincidence with the energy detector. The two fit peaks correspond to one and two photoelectrons detected. Most events are in the overflow channel (channel 1105) which is vertically off-scale.

It is also possible, however, for an electron to produce zero photoelectrons and thus go undetected. To estimate this, the single and 2 photoelectron peaks in the ADC spectrum of veto detector 6, taken with electron beam, were each fit with a Gaussian as shown in Fig. 8. The probability that *n* photoelectrons are produced is determined by the Poisson distribution,

$$P(\mu ,n)=\frac{e^{-u}\mu^{n}}{n!},$$ (1)

where *µ* is the average number of photoelectrons produced for electrons of a given energy hitting a particular position on the veto paddle. The number of events in the photoelectron peaks is then determined by the sum of these Poisson distributions over the various electron energies and paddle positions where the electrons hit. The average number of photoelectrons in the total distribution is larger than 20, so the single and two photoelectron peaks are dominated by the Poisson distributions from the lowest energy electrons and events where the light collection was poorest. So assuming these peaks can be described by a single Poisson distribution with a single *µ* and using Eq. (1)*P*(1)/*P*(2) = 2/*µ* and *P*(0)/*P*(1) = 1/*µ*. Subsequently, the Gaussians fit to the two peaks yield the number of zero photoelectrons, *P*(0) = 1790. This estimate does not include events in the tails of higher photoelectron peaks that fall under the single and two photoelectron peaks. In fact, the three photoelectron peak can be seen as a shoulder merging with the two photoelectron peak in Fig. 8. This would tend to underestimate the number of zero photoelectrons. However judging from Fig. 8, it is not unreasonable to expect the ratio *P*(1)/*P*(2) to be somewhere between 1 and 3. So somewhere between 1195 and 3585 zero photoelectron events were present. Because there were a total of 31680 coincidence events, the overall backscatter veto efficiency is estimated to be between 90 % and 96 %—within the tolerances required to produce a sub-1 % measurement of little “*a*”.

## 4. Conclusion

A prototype electron spectrometer that suppresses backscattered events was built and tested with an electron beam. The resulting data shows backscattered events were indeed rejected. The low energy tail has a component that cannot be subtracted by the veto detectors. This is thought to be due to electrons scattering on the beam transport system and from x rays produced by the electrons. The combination of a light leak in the detector and backscattered electron energies deposited in a particular veto detector indicate 90 % to 96 % detection efficiency of backscattered electrons—enough to allow a 0.5 % measurement in little “*a*”.

## Footnotes

1 Certain commercial equipment, instruments, or materials are identified in this paper to foster understanding. Such identification does not imply recommendation or endorsement by the National Institute of Standards and Technology, nor does it imply that the materials or equipment identified are necessarily the best available for the purpose.
